# The seroprevalence of West Nile Virus in Israel: A nationwide cross sectional study

**DOI:** 10.1371/journal.pone.0179774

**Published:** 2017-06-16

**Authors:** Ravit Bassal, Tamy Shohat, Zalman Kaufman, Batya Mannasse, Eilat Shinar, Doron Amichay, Mira Barak, Anat Ben-Dor, Adina Bar Haim, Daniel Cohen, Ella Mendelson, Yaniv Lustig

**Affiliations:** 1 Israel Center for Disease Control, Ministry of Health, Chaim Sheba Medical Center, Tel Hashomer, Ramat-Gan, Israel; 2 Sackler Faculty of Medicine, Tel-Aviv University, Tel-Aviv, Israel; 3 Central Virology Laboratory, Ministry of Health, Public Health Services, Chaim Sheba Medical Center, Tel Hashomer, Ramat-Gan, Israel; 4 Magen David Adom Blood Services, Israel; 5 Soroka University Medical Center, Clalit Health Services, Beer Sheva, Israel; 6 Haifa and Western Galilee Laboratories, Clalit Health Services, Nesher, Israel; 7 Schneider Children Medical Center, Clalit Health Services, Petah-Tiqwa, Israel; 8 Mayanei HaYeshua Medical Center, Bnei-Brak, Israel; CEA, FRANCE

## Abstract

West Nile Virus (WNV) is endemic in Israel, affecting yearly 40–160 individuals. Israel is located on a central migratory path between Africa and Eurasia and most West Nile Fever (WNF) cases reported in recent years were among residents of the coastal plain. The aim of the study was to evaluate the seroprevalence of WNV among the Israeli population and to assess correlates for WNV infection. A cross-sectional nationwide serologic survey was conducted using 3,145 serum samples collected by the national Israeli serum bank during 2011–2014, representing all age and population groups in Israel. Prevalence rates of WNV IgG antibodies were determined. Logistic regressions models were applied to assess the associations between demographic characteristics and WNV seropositivity. 350 samples were positive to WNV (11.1%; 95%CI: 10.0–12.3%). In the multivariable analysis, there was a significant association between seropositivity and the Arab population group vs. Jews and others (OR = 1.86, 95%CI: 1.37–2.52), the time lived in Israel [50–59 years vs. 0–9 years; OR = 10.80 (95%CI: 1.03–113.46) and ≥60 years vs. 0–9 years; OR = 14.00 (1.32–148.31)] residence area] Coastal Plain, Inland Plain (Shfela) and Great Rift Valley vs. Upper Galilee; OR = 2.24 (95%CI: 1.37–3.65), OR = 2.18 (95%CI: 1.18–4.03), OR = 1.90 (95%CI: 1.10–3.30), respectively [and rural vs. urban settlement (OR = 1.65, 95%CI: 1.26–2.16). People, who reside in the Coastal Plain, Inland Plain and Great Rift Valley, should be aware of the risk of contracting WNV and reduce exposure to mosquito bites, using insect repellents, and wearing protective clothing. The Ministry of Environmental Protection should be active in reducing the mosquito population by eliminating sources of standing water, a breeding ground for mosquitoes.

## Introduction

West Nile virus (WNV) is a mosquito-borne zoonotic arbovirus belonging to the family *Flaviviridae*, genus *Flavivirus*, and is primarily transmitted by *Culex* mosquitoes [1]. Wild birds are the natural host, while horses and humans are considered dead-end hosts. Infection with WNV is asymptomatic in most cases, while in approximately 20% it results in West Nile Fever (WNF) and in less than 1% in acute West Nile Neuroinvasive Disease (WNND) [2]. Symptoms of WNF include fever, headache, tiredness, body aches, nausea, vomiting, joint pains, occasionally with a skin rash and swollen lymph glands. WNND is characterized by signs of encephalitis, meningo-encephalitis or meningitis and is often observed among elderly [2].

Since the 1990's, several outbreaks involving high number of cases of neuroinvasive disease occurred among humans in Europe and North America [3–5]. In Israel, outbreaks due to WNV infections were reported in 1951, 1952, 1953, 1957 and 1980 [6]. WNV reemerged in Israel in the late 1990s in various avian species, including migratory storks in the south of the country, and a large outbreak which occurred in domestic geese [6]. In 2000, Israel experienced its largest recorded WNF outbreak among humans with over 400 reported cases and nearly 40 fatalities [6–9]. Since then, a total of 1,382 WNV cases were reported between 2000 and 2012 [6].

Since one of the primary routes of WNV transmission is via migrating birds [10], and the fact that Israel is located on a central migratory path between Africa and Eurasia [11], WNV infections are highly expected in Israel. Indeed, a wide variation of WNV lineages clades and clusters has been isolated from mosquitoes in Israel during the past 15 years and the Mediterranean subtype of WNV lineage 1 has been found to permanently circulate in the region [12].

Despite the high numbers of WNV infections during the last 16 years, very little is known about the WNV seroprevalence among the Israeli population. A few studies examined WNV IgG seroprevalence only in specific age groups or in distinct areas in Israel, but not in a nationwide cross-sectional survey [7, 13, 14]. In this study we evaluated the seroprevalence of WNV IgG antibodies in samples representing all age groups and assessed correlates for being positive to WNV in Israel.

## Material and methods

### Study design

A cross-sectional study was performed using a sample of stored sera that had been collected for the national Israeli sera bank at the Israeli Center for Disease Control which was established in 1997. Samples were residuals from diagnostic laboratories and healthy blood donors. Sera from subjects with confirmed or suspected immunological disorders were discarded. For each serum sample, basic demographic information was recorded at the time of specimen collection. Variables included patient age, gender, place of residence (city), birth country, population group ("Jews and others" included Jews, non-Arabic Christians and population not affiliated with a religion; "Arabs" included Muslims, Arab Christians and Druze) and the date in which the sample was drawn, using computerized records from the laboratories. The socio-economic status was allocated to each participant on the basis of the given address using the socio-economic residential classification [15] and was divided to low (1–5) vs. high (6–10). The permanently inhabited regions in Israel were divided into urban (≥10,000 residents) and rural (<10,000 residents) areas. The sera were stored at -80°C until separation and laboratory analysis.

### Sampling

Israel was divided into seven geographical areas: Upper Galilee, Lower Galilee, South, Jerusalem, Coastal Plain, The Inland Plain (Shfela) and The Great Rift Valley. Sample size was calculated by the expected proportion of positive samples, based on previous studies performed in Israel using 80% power at the 0.05 level of significance (two-sided) (maximum 300 samples). In total, 3,145 samples were included, using sera collected for the national Israeli sera bank between 2011 and 2014 (349, 469, 815, 382, 596, 222 and 312 samples were retrieved from Upper Galilee, Lower Galilee, South, Jerusalem, Coastal Plain, The Inland Plain and The Great Rift Valley, respectively).

### Laboratory testing

WNV Immunoglobulin G (IgG) levels were determined using a commercial ELISA kit (IgG DxSelect ELISA, Focus Diagnostics Inc., Cypress, CA), according to the manufacturer’s instructions. The assay was performed by the Central Virology Laboratory of the Ministry of Health. Samples presenting an IgG index value (O.D. level divided by the O.D. of the cut-off) of 1.5 and above were considered positive and with an index value of less than 1.3 were considered negative. Samples with an IgG index value ≥1.3 and less than 1.5 were considered borderline. All samples with borderline results and a subset of samples with positive result were further analyzed by a serum neutralization test with WNV and Usutu virus, where a titer of 1:10 or more obtained was considered positive [16].

### Ethics statement

Sera collection was approved by the legal department of the Israeli Ministry of Health.

### Data analysis

Prevalence rates of WNV IgG antibodies were calculated by dividing the number of samples positive to WNV by the number of samples tested, overall, by age group, population group, gender, residence area, socio-economic status, number of years lived in Israel (for those born abroad, the number of years since arriving to Israel; and for those born in Israel, the number of years since birth), birth country and type of residence. A logistic regression model was applied to assess the association between demographic characteristics and WNV seropositivity, using the odds ratios and 95% Confidence Interval (CI) as measure of association. Multivariable analysis included variables which were significantly associated with seropositivity in the univariable analysis. Effect modification and interaction were assessed for each covariate associated with seropositivity. Statistical significance was evaluated using 2-sided tests with an alpha level of 0.05. All analyses were performed using SAS software package (version 9.1.3, SAS Institute Inc., Cary, NC, USA).

## Results

A total of 3,145 serum samples were tested for the presence of WNV IgG antibodies using an ELISA assay. Table 1 presents the demographic characteristics of the study participants.

**Table 1:** Demographic characteristics of the study participants (N = 3,145).

		N	%
**Age (years)**	**0–9**	886	28.2
	**10–29**	941	29.9
	**30–54**	476	15.1
	**55–64**	388	12.3
	**65+**	454	14.4
**Population group**	**Jews and others**	2,591	82.5
	**Arabs**	549	17.5
**Gender**	**Male**	1,623	51.6
	**Female**	1,522	48.4
**Number of years lived in Israel (years)**	**0–9**	912	29.0
	**10–19**	695	22.1
	**20–29**	340	10.8
	**30–39**	222	7.1
	**40–49**	205	6.5
	**50–59**	310	9.9
	**≥60**	461	14.7
**Birth country**	**Israel**	2,529	80.4
	**Other**	615	19.6
**Socioeconomic status**	**Low**	1,642	67.5
	**High**	789	32.5
**Residence area**	**Upper Galilee**	349	11.1
	**Lower Galilee**	469	14.9
	**South**	815	25.9
	**Jerusalem**	382	12.2
	**Coastal Plain**	596	19.0
	**The Inland Plain**	222	7.1
	**Great Rift Valley**	312	9.9
**Residence type**	**Urban**	963	30.7
	**Rural**	2,175	69.3

Results show that 350 samples were positive for WNV IgG antibodies in the ELISA test (S1 Table), suggesting past WNV infection. In order to examine the probability of false positive results due to cross-reactivity or lack of specificity, neutralization assay was performed on 134 IgG positive samples [100 samples with low positive results (Positive/cut-off ratio of 1.5–2.5) and 34 samples with high positive results (Positive/cut-off ratio of 4.5–5)] in the ELISA assay with WNV and Usutu virus. All high positive and only 22 low positive samples neutralized WNV (S1 Table). All samples tested did not neutralize Usutu virus. In order to measure the differential sensitivities for both neutralization and ELISA assays, 4 high positive samples which neutralized WNV at dilutions of 1:80–1:320 were subjected to WNV IgG ELISA at dilutions of 1:200 to 1:3200. Results show that all samples were positive for WNV IgG at substantially higher dilutions than the neutralization assay (S2 Table).

**Fig 1:**
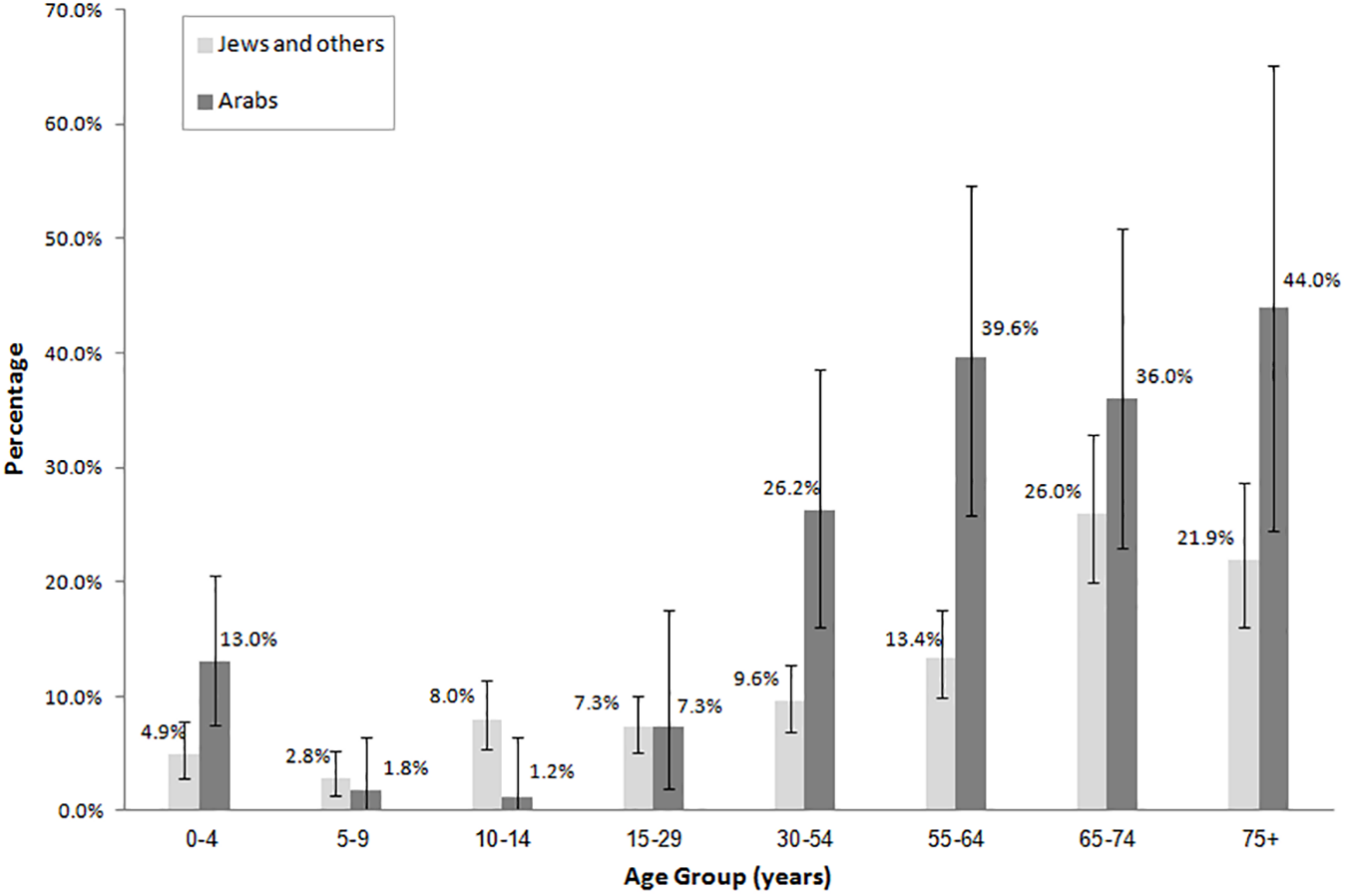
Seroposivity of IgG antibodies against WNV in Jews and others and in Arabs by age group.

Fig 1 describes the seroprevalence among Jews and others and among Arabs, by age group. In ages 0–4 years, the seroprevalence was higher among Arabs (13.0%) than among Jews and others (4.9%). High seroprevalence was found in people of older age both in Jews and others (26.0% (95%CI: 20.0–32.8%) in ages 65–74 and 21.9% (95%CI: 16.1–28.6%) in ages 75 and above) and in Arabs (36.0% (95%CI: 22.9–50.8%) in ages 65–74 and 44.0% (95%CI: 24.4–65.1%) in ages 75 and above).

The overall seroprevalence of WNV of the samples tested was 11.1%; 95%CI: 10.0–12.3%. Table 2 presents the seroprevalence of WNV IgG antibodies by demographic characteristics. WNV seropositivity was significantly associated with higher age (30–54, 55–64 and ≥65 vs. 0–9 years; OR = 2.61 (95%CI: 1.73–3.96), OR = 3.94 (95%CI: 2.63–5.92), OR = 7.04 (4.86–10.20), respectively), being Arabs (vs. Jews and others) (OR = 1.67, 95%CI: 1.28–2.17), being male (vs. female) (OR = 1.29, 95%CI: 1.03–1.61), higher time of residency in Israel (30–39, 40–49, 50–59 and ≥60 vs. 0–9 years; OR = 2.28 (95%CI: 1.35–3.86), OR = 3.38 (95%CI: 2.07–5.53), OR = 4.16 (2.73–6.34), OR = 7.92 (95%CI: 5.50–11.39), respectively), being born abroad (OR = 1.37, 95%CI: 1.06–1.78), residence area (Lower Galilee, Coastal Plain and Great Rift Valley vs. Upper Galilee; OR = 1.68 (95%CI: 1.04–2.72), OR = 2.04 (95%CI: 1.30–3.21), OR = 1.86 (95%CI: 1.11–3.09), respectively) and settlement type (rural vs. urban) (OR = 1.66, 95%CI: 1.32–2.08).

**Table 2:** Seroprevalence and univariate logistic regression analysis of associated possible correlates for West Nile virus in subjects in Israel.

		Positive				
		N	%	OR	95%CI	p-value
**Age (years)**	0–9	43	4.8	Ref.		
	10–29	66	7.0	1.48	1.00–2.20	0.05
	30–54	56	11.8	2.61	1.73–3.96	<0.01
	55–64	65	16.8	3.94	2.63–5.92	<0.01
	65+	120	26.4	7.04	4.86–10.20	<0.01
**Population group**	Jews and others	263	10.2	Ref.		
	Arabs	87	15.8	1.67	1.28–2.17	<0.01
**Gender**	Female	150	9.9	Ref.		
	Male	200	12.3	1.29	1.03–1.61	0.03
**Number of years lived in Israel (years)**	0–9	44	4.8	Ref.		
	10–19	45	6.5	1.37	0.89–2.10	0.15
	20–29	22	6.5	1.36	0.80–2.31	0.25
	30–39	23	10.4	2.28	1.35–3.86	<0.01
	40–49	30	14.6	3.38	2.07–5.53	<0.01
	50–59	54	17.4	4.16	2.73–6.34	<0.01
	≥60	132	28.6	7.92	5.50–11.39	<0.01
**Birth country**	Israel	265	10.5	Ref.		
	Other	85	13.8	1.37	1.06–1.78	0.02
**Socioeconomic status**	Low	151	9.2	Ref.		
	High	88	11.2	1.24	0.94–1.64	0.13
**Residence area**	Upper Galilee	27	7.7	Ref.		
	Lower Galilee	58	12.4	1.68	1.04–2.72	0.03
	South	83	10.2	1.35	0.86–2.13	0.19
	Jerusalem	29	7.6	0.98	0.57–1.69	0.94
	Coastal Plain	87	14.6	2.04	1.30–3.21	<0.01
	The Inland Plain	24	10.8	1.45	0.81–2.58	0.21
	Great Rift Valley	42	13.5	1.86	1.11–3.09	0.02
**Residence type**	Urban	207	9.5	Ref.		
	Rural	143	14.8	1.66	1.32–2.08	<0.01

OR: Odds Ratio; CI: Confidence Interval.

According to the multivariable analysis, high risk for being WNV IgG seropositive was observed among Arabs vs. Jews and others; OR = 1.86, 95%CI: 1.37–2.52, higher number of years lived in Israel (50–59 and ≥60 vs. 0–9 years; OR = 10.80 (95%CI: 1.03–113.46), OR = 14.00 (1.32–148.31), respectively), residence area (Coastal plain, The Inland Plain and Great Rift Valley vs. Upper Galilee; OR = 2.24 (95%CI: 1.37–3.65), OR = 2.18 (95%CI: 1.18–4.03), OR = 1.90 (95%CI: 1.10–3.30), respectively) and rural settlement (OR = 1.65, 95%CI: 1.26–2.16) (Table 3).

**Table 3:** Multivariate logistic regression analysis of associated possible correlates for West Nile virus in subjects in Israel.

		OR	95%CI	p-value
**Age (years)**	**0–9**	Ref.		
	**10–29**	1.73	0.21–14.10	0.61
	**30–54**	0.39	0.04–4.18	0.43
	**55–64**	0.38	0.03–4.12	0.42
	**65+**	0.66	0.06–7.28	0.73
**Population group**	**Jews and others**	Ref.		
	**Arabs**	1.86	1.37–2.52	<0.01
**Gender**	**Female**	Ref.		
	**Male**	1.26	0.99–1.59	0.06
**Number of years lived in Israel (years)**	**0–9**	Ref.		
	**10–19**	0.89	0.11–7.17	0.91
	**20–29**	0.96	0.12–7.82	0.97
	**30–39**	5.73	0.53–61.81	0.15
	**40–49**	8.30	0.78–88.30	0.08
	**50–59**	10.80	1.03–113.46	<0.05
	**≥60**	14.00	1.32–148.31	0.03
**Birth country**	**Israel**	Ref.		
	**Other**	1.06	0.75–1.49	0.74
**Residence area**	**Upper Galilee**	Ref.		
	**Lower Galilee**	1.55	0.94–2.56	0.09
	**South**	1.43	0.89–2.30	0.14
	**Jerusalem**	1.27	0.70–2.29	0.43
	**Coastal Plain**	2.24	1.37–3.65	<0.01
	**The Inland Plain**	2.18	1.18–4.03	0.01
	**Great Rift Valley**	1.90	1.10–3.30	0.02
**Residence type**	**Urban**	Ref.		
	**Rural**	1.65	1.26–2.16	<0.01

OR: Odds Ratio; CI: Confidence Interval

## Discussion

WNV has been the cause of several outbreaks of WNF in Europe, the Middle East and the Americas in the last 20 years [17]. Since most WNV infections are asymptomatic, a seroprevalence survey was needed to examine the exposure of the population to the virus and to identify areas with high endemicity. In this study we performed a large nationwide cross sectional study to determine the seroprevalence of WNV antibodies among the Israeli population.

The results of this survey revealed an overall seroprevalence of 11.1%, higher than found in previous serosurveys performed in other endemic countries such as Greece (2.1%) [18], Turkey (2.5%) [19], New-York (2.6%) [20] and Spain (6.5%) [21], but lower than found in Gabon, residing on the west coast of Central Africa (27.2%) [22]. The high IgG seroprevalence in the present study suggests high virus circulation in Israel. Previous studies performed in Israel in the late 1990's presented lower seroprevalence than this study [7, 13, 14]. An exception is a study performed on 285 healthy soldiers aged 40–55 years where 41.9% were IgG positive [14]. Indeed, since 2000 Israel experienced high number of WNV cases [6] and substantial circulation of WNV in mosquitoes is found each year [12]. The high seroprevalence detected herewith is not likely due to cross reactivity since other flaviviruses infecting humans are uncommon in Israel. Indeed, no neutralization of Usutu virus was detected in all 134 positive WNV ELISA IgG samples.

One of the most interesting observations made by this study is that the number of years lived in Israel was associated with being WNV infected. Evidence of high exposure among older people was found in previous studies in Israel [7, 13, 14] and in other countries [18, 22]. However, using the number of years lived in Israel has allowed us to differentiate between being old and spending more time in Israel. Our results show that the high seroprevalence in older people is not due to age but due to the longer period of time spent in Israel, which reflects the cumulative time of exposure during life to outdoor activities and the risk of being exposed to infected mosquitoes.

Our results show that the seroprevalence among Arabs was significantly higher compared with Jews. To the best of our knowledge, this is the first report describing this difference. Our results may be explained by the lifestyle disparities between these two populations. In Israel, the Arab population is at lower socio-economic status than Jews [23], and mostly resides in separate settlements located in rural areas where standing water and open sewage systems might be close by. In correlation with this finding, we found that those living in the rural localities had higher seroprevalence than people living in cities. This is not surprising since people who live, work or undertakes recreational activities in rural, agricultural or horticultural settings are at higher risk of mosquito borne infections [24]. Since mosquitoes thrive in these environmental conditions, and more Arabs reside in rural areas than Jews, it is not unconceivable to hypothesize that Arabs are at higher risk of WNV infection. The high seroprevalence we have shown among the young Arab population supports the assumptions that outdoor activities, which is higher among the lower age group, and environmental conditions are both associated with higher probability to be exposed to mosquito bites.

Our study shows significant differences in WNV seroprevalence among geographic regions within Israel as has also been shown for other countries such as the state of Nabraska and Greece [18, 25]. Israeli citizens residing in the Coastal Plain, The Inland Plain and the Great Rift Valley had the highest seroprevalence of WNV antibodies, as compared with those living in Jerusalem, Upper Galilee and the South. Interestingly, these findings correlate well with the incidence of West Nile Fever cases in Israel. During the 2000 outbreak, and since then, WNV infections in humans have consistently been concentrated along Israel’s densely populated Coastal Plain, from north of Haifa to south of Tel Aviv, while sporadic cases were far fewer in the Negev desert, and in Jerusalem [6, 9, 26]. The high seroprevalence in the Coastal Plain may be explained by the appearance of WNV infected mosquitoes observed in catches from altitude<300 meters [27]. We have also demonstrated high variation in the seroprevalence within Israel and Wimberly et al (2014) have explained the geographical differences of WNV outbreaks within areas in the United States by the variation in temperatures and precipitation [28], which could also play a role in our case, as Israel is part of the birds migration routes and as a consequence, viral distribution may also have a part in the observed geographic variation in Israel.

Our study is based on the results of an IgG ELISA assay which was shown to have 97.0% specificity among “naïve” blood donor population [29]. Our results suggest that all 34 high positive and only 22 of 100 low positive ELISA IgG samples neutralized WNV, however that for 4 high positive IgG ELISA samples, the sensitivity of the ELISA IgG far exceeds the sensitivity of the neutralization. Therefore, the discrepancy between the neutralization and ELISA assays in diagnosing past WNV infection plausibly stems from differences in sensitivity and not due to cross-reactivity or lack in specificity of the ELISA IgG. This may be especially noticeable in seroprevalence studies that examine the general population which may have been exposed to WNV many years ago and therefore have low levels of IgG antibodies that are detected in the ELISA assay but are unable to neutralize WNV. We cannot exclude the possibility that some samples that tested positive by the ELISA are false positive due to cross-reactivity or lack of specificity, however, our results as well as the performance of the ELISA assay [29] suggest that most of the ELISA IgG positive samples are true positives and therefore were included in the study.

Although this study has tested a high number of samples with a broad representation of the Israeli population, we should keep in mind that the lack of information regarding the actual exposure to mosquitoes which is an important aspect of WNV seroprevalence and the reliance on the ELISA IgG assay which may cause overestimation of the real seroprevalence in Israel are limitations of this study.

In conclusion, after several years with frequent human infections by WNV, we found high (11.1%) seroprevalence of WNV in Israel in a nationwide cross sectional study. Our results show that the numbers of years lived in Israel and living in the coastal region of Israel are both associated with higher seroprevalence. The difference in WNV seroprevalence in different regions within Israel emphasizes the diverse risk within Israel of being infected with WNV. Since no WNV vaccine is available for humans, the most effective way to avoid WNV infections is to raise the awareness of the population on reducing the risk of mosquito bites by using insect repellents, wearing protective clothing and reducing mosquito breeding sites. At the national level, the mosquito surveillance program established by The Ministry of Environmental Protection should minimize the transmission of the virus all over Israel, but should focus especially at high risk areas.

## Supporting information

S1 Table Results of WNV ELISA IgG assay for 350 samples and WNV neutralization for 134 samples. (DOCX)

S2 Table Differential sensitivities of ELISA and neutralization WNV assays. (DOCX)
