# Supplementary material for: The seroprevalence of West Nile Virus in Israel: A nationwide cross sectional study
Source: PLoS One. 2017 Jun 16;12(6):e0179774. doi: 10.1371/journal.pone.0179774 (PMC5473576; doi:10.1371/journal.pone.0179774)
Supplement: S1 Table — (DOCX) [file pone.0179774.s001.docx]

**S1 Table.** **Results of WNV ELISA IgG assay for 350 samples and WNV neutralization for 134 samples**

| **Neutralization result (titer)** | **Elisa IgG result (OD/Cut-off ratio)** | **Sample Number** |
| --- | --- | --- |
|  | 3.93 | 11 |
|  | 3.64 | 12 |
|  | 3.33 | 19 |
|  | 4.06 | 23 |
|  | 3.25 | 24 |
|  | 3.79 | 26 |
|  | 3.81 | 29 |
| 1:80 | 2.56 | 50 |
|  | 3.61 | 51 |
|  | 3.24 | 67 |
| 1:80 | 2.14 | 73 |
|  | 3.13 | 76 |
|  | 3.12 | 84 |
|  | 3.27 | 86 |
|  | 3.53 | 93 |
|  | 4.36 | 98 |
|  | 4.23 | 102 |
|  | 4.36 | 104 |
|  | 4.23 | 114 |
|  | 3.02 | 119 |
|  | 2.83 | 126 |
|  | 3.66 | 136 |
|  | 4.07 | 141 |
|  | 3.84 | 146 |
|  | 3.3 | 153 |
|  | 3.59 | 170 |
|  | 3.05 | 172 |
|  | 2.97 | 179 |
| No | 1.78 | 182 |
| No | 1.54 | 194 |
|  | 4.4 | 197 |
| No | 1.53 | 213 |
| 1:80 | 3.78 | 257 |
|  | 3.11 | 258 |
|  | 3.62 | 259 |
|  | 3.94 | 265 |
|  | 4.2 | 269 |
|  | 4.35 | 270 |
|  | 4.41 | 274 |
|  | 4.15 | 283 |
|  | 4.54 | 292 |
|  | 4.14 | 293 |
|  | 3.6 | 315 |
|  | 3.39 | 339 |
|  | 4.76 | 345 |
|  | 3.21 | 346 |
|  | 3.01 | 395 |
|  | 3.92 | 407 |
| No | 2.29 | 416 |
|  | 3.02 | 444 |
| No | 2.14 | 459 |
|  | 4.18 | 461 |
|  | 3.35 | 465 |
|  | 3.63 | 472 |
|  | 3.67 | 473 |
|  | 3.94 | 476 |
|  | 3.55 | 482 |
|  | 3.51 | 487 |
| No | 1.79 | 488 |
|  | 3.78 | 495 |
|  | 2.84 | 500 |
|  | 4.2 | 502 |
|  | 3.66 | 522 |
| No | 1.63 | 528 |
|  | 3.4 | 533 |
| No | 1.65 | 549 |
|  | 4.43 | 559 |
|  | 3.88 | 567 |
|  | 3.01 | 578 |
|  | 2.77 | 580 |
|  | 3.58 | 586 |
|  | 3.49 | 587 |
| No | 2.19 | 617 |
| No | 1.66 | 618 |
|  | 3.6 | 620 |
|  | 4.15 | 635 |
| No | 2.13 | 649 |
| No | 1.53 | 660 |
|  | 3.91 | 677 |
| 1:40 | 4.63 | 680 |
| No | 1.69 | 681 |
|  | 4.48 | 684 |
|  | 4.13 | 687 |
|  | 4.08 | 688 |
|  | 4.34 | 696 |
| No | 1.79 | 698 |
| No | 1.64 | 711 |
|  | 4.46 | 718 |
|  | 4.16 | 728 |
|  | 5.13 | 731 |
|  | 3.84 | 771 |
|  | 2.67 | 775 |
|  | 4.33 | 803 |
|  | 3.9 | 812 |
|  | 4.4 | 817 |
| No | 1.79 | 819 |
|  | 4.14 | 859 |
| 1:10 | 1.72 | 876 |
| 1:40 | 4.69 | 878 |
|  | 4.21 | 882 |
|  | 4.29 | 895 |
| 1:80 | 4.7 | 897 |
|  | 4.39 | 899 |
|  | 4.59 | 900 |
|  | 3.82 | 902 |
|  | 3.94 | 904 |
|  | 4.43 | 911 |
|  | 4.29 | 921 |
|  | 2.84 | 927 |
|  | 3.35 | 936 |
| No | 1.63 | 989 |
| No | 2.41 | 990 |
|  | 3.26 | 1008 |
|  | 3.78 | 1009 |
| 1:10 | 2.12 | 1013 |
| 1:20 | 2.59 | 1034 |
| No | 1.86 | 1036 |
| No | 1.56 | 1063 |
|  | 5.48 | 1072 |
|  | 5.42 | 1078 |
| No | 2.33 | 1086 |
| 1:160 | 4.72 | 1100 |
| 1:40 | 4.83 | 1106 |
|  | 4 | 1108 |
| No | 1.79 | 1115 |
|  | 4.35 | 1131 |
|  | 5.18 | 1133 |
| No | 1.84 | 1136 |
| 1:40 | 4.65 | 1140 |
| 1:320 | 4.84 | 1144 |
|  | 3.91 | 1151 |
|  | 4.5 | 1153 |
|  | 4.55 | 1156 |
|  | 3.56 | 1157 |
| 1:10 | 2.09 | 1165 |
| 1:40 | 4.83 | 1173 |
|  | 3.83 | 1183 |
|  | 3.75 | 1184 |
|  | 3.08 | 1185 |
|  | 4.55 | 1192 |
|  | 3 | 1200 |
| No | 1.78 | 1219 |
|  | 4.2 | 1221 |
|  | 4.3 | 1225 |
|  | 2.89 | 1238 |
|  | 3.81 | 1241 |
| No | 1.85 | 1246 |
|  | 4.34 | 1247 |
| 1:160 | 4.82 | 1259 |
| 1:20 | 4.59 | 1261 |
| 1:10 | 1.66 | 1264 |
|  | 4.56 | 1273 |
|  | 2.96 | 1279 |
|  | 3.75 | 1282 |
| 1:160 | 4.86 | 1289 |
|  | 3.66 | 1299 |
| 1:10 | 2.55 | 1312 |
|  | 3.49 | 1324 |
| No | 1.55 | 1328 |
| 1:10 | 1.83 | 1346 |
| No | 1.75 | 1357 |
|  | 3.41 | 1374 |
|  | 2.83 | 1387 |
| No | 1.53 | 1396 |
| No | 1.66 | 1399 |
| 1:20 | 1.98 | 1401 |
| 1:20 | 2.03 | 1403 |
|  | 3.7 | 1420 |
| No | 1.52 | 1426 |
|  | 3.03 | 1435 |
|  | 4.44 | 1438 |
|  | 3.12 | 1444 |
|  | 2.87 | 1457 |
| 1:160 | 4.63 | 1479 |
| No | 1.57 | 1484 |
| No | 1.97 | 1485 |
|  | 4.23 | 1498 |
| No | 2.03 | 1517 |
| No | 1.98 | 1521 |
| 1:160 | 4.59 | 1542 |
| No | 2.19 | 1595 |
| No | 1.57 | 1599 |
| No | 2.3 | 1615 |
| 1:10 | 1.59 | 1630 |
| 1:10 | 1.52 | 1636 |
| No | 2.11 | 1642 |
|  | 4.02 | 1655 |
|  | 3.69 | 1669 |
|  | 3.74 | 1690 |
| No | 2.13 | 1701 |
|  | 4.52 | 1708 |
| No | 1.81 | 1711 |
| 1:80 | 4.92 | 1713 |
|  | 5.16 | 1716 |
|  | 2.67 | 1717 |
|  | 4.15 | 1730 |
| 1:320 | 5 | 1731 |
|  | 4.58 | 1739 |
| No | 1.65 | 1745 |
|  | 3.25 | 1746 |
|  | 3.01 | 1747 |
|  | 3.73 | 1751 |
|  | 3.98 | 1752 |
| 1:320 | 4.93 | 1771 |
|  | 4.54 | 1794 |
| 1:40 | 2.61 | 1802 |
| No | 1.7 | 1805 |
| 1:160 | 4.85 | 1814 |
| No | 1.86 | 1816 |
|  | 3.76 | 1818 |
|  | 4.11 | 1823 |
|  | 4.02 | 1825 |
|  | 3.87 | 1835 |
|  | 4.27 | 1842 |
|  | 4.54 | 1847 |
| No | 2.54 | 1854 |
| No | 2.22 | 1858 |
|  | 4.4 | 1861 |
| No | 1.98 | 1863 |
| 1:320 | 4.97 | 1866 |
|  | 3.18 | 1875 |
| No | 1.55 | 1877 |
|  | 4.53 | 1889 |
|  | 3.37 | 1891 |
|  | 5.28 | 1892 |
| No | 1.94 | 1899 |
| 1:640 | 4.68 | 1904 |
| 1:160 | 4.84 | 1907 |
| No | 1.51 | 1911 |
|  | 4.27 | 1913 |
|  | 5.01 | 1917 |
| 1:80 | 4.97 | 1920 |
| 1:40 | 4.76 | 1928 |
|  | 4.17 | 1930 |
|  | 2.78 | 1937 |
| 1:160 | 4.76 | 1940 |
|  | 5.06 | 1945 |
| 1:80 | 1.91 | 1970 |
|  | 3.07 | 1984 |
|  | 4.31 | 2051 |
|  | 4.5 | 2052 |
|  | 4.41 | 2060 |
| No | 1.52 | 2086 |
| 1:160 | 4.62 | 2099 |
| No | 2.29 | 2134 |
|  | 2.85 | 2136 |
| No | 2.29 | 2149 |
|  | 2.75 | 2156 |
| No | 1.98 | 2158 |
| No | 1.69 | 2163 |
| No | 1.92 | 2166 |
| No | 2.26 | 2178 |
|  | 4.49 | 2180 |
|  | 3.65 | 2183 |
| No | 1.62 | 2185 |
|  | 6.96 | 2193 |
| No | 1.56 | 2198 |
| No | 2.07 | 2209 |
| No | 1.85 | 2211 |
| No | 2.16 | 2212 |
| No | 2.62 | 2213 |
| 1:10 | 1.91 | 2214 |
|  | 4.4 | 2215 |
| No | 2.1 | 2228 |
|  | 5.37 | 2260 |
|  | 3.17 | 2267 |
| 1:160 | 4.77 | 2336 |
|  | 4.14 | 2339 |
|  | 3.77 | 2346 |
|  | 4.55 | 2353 |
|  | 3.31 | 2366 |
|  | 4.15 | 2384 |
| 1:320 | 4.98 | 2405 |
| 1:10 | 2.66 | 2414 |
|  | 5.59 | 2429 |
|  | 3.24 | 2442 |
|  | 5.61 | 2444 |
|  | 5.21 | 2447 |
|  | 2.94 | 2450 |
|  | 5.36 | 2456 |
|  | 6.11 | 2457 |
| No | 1.61 | 2468 |
|  | 4.01 | 2471 |
|  | 5.64 | 2480 |
|  | 2.9 | 2484 |
|  | 6.32 | 2511 |
| 1:10 | 1.59 | 2517 |
|  | 5.18 | 2539 |
|  | 5.74 | 2544 |
|  | 4.95 | 2552 |
|  | 5.72 | 2559 |
|  | 6.34 | 2583 |
| No | 2.37 | 2587 |
|  | 3.64 | 2603 |
|  | 4.28 | 2617 |
|  | 4.06 | 2621 |
|  | 4.31 | 2622 |
|  | 3.75 | 2648 |
|  | 4.32 | 2649 |
|  | 3.76 | 2656 |
|  | 3.96 | 2675 |
| 1:160 | 5.02 | 2676 |
| 1:160 | 4.68 | 2677 |
|  | 3.3 | 2689 |
|  | 4.48 | 2697 |
|  | 3.7 | 2702 |
|  | 4.4 | 2703 |
| 1:160 | 4.67 | 2704 |
|  | 3.03 | 2716 |
| 1:40 | 1.84 | 2725 |
|  | 3.86 | 2740 |
| 1:640 | 2.65 | 2755 |
|  | 5.12 | 2765 |
|  | 5.17 | 2773 |
|  | 3.72 | 2806 |
|  | 5.04 | 2811 |
| 1:160 | 4.97 | 2818 |
| 1:10 | 4.96 | 2844 |
|  | 6.08 | 2857 |
| 1:40 | 4.84 | 2859 |
|  | 6.02 | 2870 |
|  | 5.82 | 2873 |
|  | 4.01 | 2890 |
| No | 1.73 | 2896 |
| No | 1.6 | 2900 |
| No | 1.87 | 2908 |
|  | 4.12 | 2913 |
| No | 2.33 | 2925 |
|  | 5.35 | 2929 |
| No | 2.68 | 2932 |
|  | 4.49 | 2949 |
| 1:80 | 4.69 | 2950 |
|  | 5.42 | 2952 |
|  | 4.38 | 2973 |
| 1:10 | 2.63 | 2976 |
|  | 4.55 | 2980 |
|  | 4.16 | 2986 |
| No | 2.29 | 3010 |
|  | 4.41 | 3014 |
|  | 4.37 | 3026 |
|  | 4.06 | 3028 |
|  | 5.18 | 3030 |
| 1:10 | 2.25 | 3033 |
| No | 2.4 | 3036 |
| No | 2.55 | 3038 |
| No | 1.75 | 3044 |
| No | 1.58 | 3048 |
| No | 1.9 | 3065 |
| No | 1.91 | 3093 |
| No | 2.61 | 3103 |
