# Supplementary material for: The seroprevalence of West Nile Virus in Israel: A nationwide cross sectional study
Source: PLoS One. 2017 Jun 16;12(6):e0179774. doi: 10.1371/journal.pone.0179774 (PMC5473576; doi:10.1371/journal.pone.0179774)
Supplement: S2 Table — (DOCX) [file pone.0179774.s002.docx]

**S2 Table. Differential sensitivities of ELISA and neutralization WNV assays**

| **Sample Number** | **Positive neutralization dilution factor** | **Positive ELISA dilution factor** |
| --- | --- | --- |
| 1 | 1:80 | 1:1600 |
| 2 | 1:160 | 1:400 |
| 3 | 1:160 | 1:800 |
| 4 | 1:320 | 1:1600 |
